# Sulfatide Regulates Caspase-3-Independent Apoptosis of Influenza A Virus through Viral PB1-F2 Protein

**DOI:** 10.1371/journal.pone.0061092

**Published:** 2013-04-04

**Authors:** Tadanobu Takahashi, Masahiro Takaguchi, Tatsuya Kawakami, Takashi Suzuki

**Affiliations:** Department of Biochemistry, School of Pharmaceutical Sciences, University of Shizuoka, Suruga-ku, Shzuoka, Japan; National Institute for Viral Disease Control and Prevention, CDC, China

## Abstract

Influenza A virus (IAV) generally causes caspase-dependent apoptosis based on caspase-3 activation, resulting in nuclear export of newly synthesized viral nucleoprotein (NP) and elevated virus replication. Sulfatide, a sulfated galactosylsphingolipid, enhances IAV replication through promoting newly synthesized viral NP export induced by association of sulfatide with hemagglutinin delivered to the cell surface. Here, we demonstrated that sulfatide is involved in caspase-3-independent apoptosis initiated by the PB1-F2 protein of IAV by using genetically sulfatide-produced cells and PB1-F2-deficient IAVs. Sulfatide-deficient COS7 cells showed no virus-induced apoptosis, whereas SulCOS1 cells, sulfatide-enriched COS7 cells that genetically expressed the two transferases required for sulfatide synthesis from ceramide, showed an increase in IAV replication and were susceptible to caspase-3-independent apoptosis. Additionally, PB1-F2-deficient IAVs, which were generated by using a plasmid-based reverse genetics system from a genetic background of A/WSN/33 (H1N1), demonstrated that PB1-F2 contributed to caspase-3-independent apoptosis in IAV-infected SulCOS1 cells. Our results show that sulfatide plays a critical role in efficient IAV propagation via caspase-3-independent apoptosis initiated by the PB1-F2 protein.

## Introduction

Influenza A virus (IAV) often causes serious respiratory injuries and worldwide outbreaks in many mammalian and avian species including humans. IAV induces caspase-dependent apoptosis through caspase-3 activation [1] provoked by viral proteins such as neuraminidase (NA) [2], nonstructural protein 1 (NS1) [3], PB1-F2 [4], and hemagglutinin (HA) [5], resulting in increased virus replication owing to enhanced export of newly synthesized viral nucleoprotein (NP) from the nucleus to the cytosol [6]. On the other hand, IAV propagation is impaired by inhibition of the Raf/MEK/ERK signaling cascade that results in nuclear retention of viral ribonucleoprotein (vRNP) complexes [7]. Furthermore, membrane accumulation of HA triggers nuclear export of the viral genome via protein kinase C alpha-mediated activation of ERK signaling [8].

Sulfatide is one of the major sulfated glycolipids detected in lipid rafts of plasma membranes, various mammalian organs including the brain, kidney, respiratory tract and gastrointestinal tract, and cell lines of mammalian kidneys, which are used for the primary isolation and cultivation of IAV. We showed that IAV binds to sulfatide [9] and that sulfatide enhances IAV replication through promoting nascent viral NP export induced by association with HA delivered to the cell surface [10], [11]. However, how sulfatide is associated with viral replication remains unknown.

Although virus-induced apoptosis is thought to be the initiation step of host defense prior to antigen presentation, it remains unknown whether virus-induced apoptosis works in an advantageous or disadvantageous way for the virus itself. For IAV, it has been suggested that virus-induced apoptosis via caspase-3 activation is advantageous for virus replication by promoting translocation of the newly synthesized vRNP from the nucleus to cytoplasm [6].

In this study, we investigated the effect of sulfatide expression on IAV-induced apoptosis. IAV induced caspase-3-independent apoptosis in sulfatide-enriched SulCOS1 cells. This cell line is generated by introduction of two transferases, ceramide galactosyltransferase and cerebroside sulfotransferase, into COS7 cells, a sulfatide-deficient cell line [10], [12]. These transferases are required for sulfatide synthesis. IAV-induced caspase-3 activation was not observed in SulCOS1 cells. Apoptosis-inducing factor (AIF) was translocated from mitochondria to the nucleus in SulCOS1 cells, indicating a hallmark of caspase-3-independent apoptosis [13]. Furthermore, PB1-F2 (a frame-shift protein from the PB1 gene of IAV), which is known to localize at mitochondria, functioned as an inducer of sulfatide-associated caspase-3-independent apoptosis through this translocation of AIF. Sulfatide expression enhanced virus replication through caspase-3-independent apoptosis.

## Materials and Methods

### Cells and viruses

Madin-Darby canine kidney (MDCK) cells were maintained in Eagle's minimum essential medium (MEM) supplemented with 5% fetal bovine serum (FBS). COS7 cells and SulCOS1 cells [10] were maintained in Dulbecco's modified MEM supplemented with 10% FBS. IAV A/WSN/33 (H1N1) strain was propagated and purified as described previously [14]. Two PB1-F2-deficient mutant viruses and wild-type virus with a backbone of WSN were generated using a plasmid-driven reverse genetics system. These viruses were propagated in the presence of acetylated trypsin (2 µg/ml) in MDCK cells.

### Flowcytometric analysis of virus-induced apoptosis

Cells were infected with IAV at a multiplicity of infection (MOI) of 2 plaque-forming units (PFU) per cell for 1 h at 34°C. The infected cells were maintained in a medium containing 20 µM cyclosporin A (CycA; BIOMOL Research Laboratories Inc., Plymouth Meeting, PA), 50 µM Z-VAD-FMK (VAD; R & D Systems Inc., Minneapolis, MN), culture supernatant of mouse anti-sulfatide monoclonal antibody (GS-5), or mouse anti-Gb_3_Cer monoclonal antibody (TU-1) [10] supplemented with 5% FBS at 34°C and were harvested by treatment with 0.125% trypsin at 24 h postinfection. As a control, a medium without any antibodies or inhibitors supplemented with 5% FBS was used. Phosphatidylserine externalization that resulted from virus-induced apoptosis was examined with a two color analysis of fluorescein isothiocyanate (FITC)-conjugated annexin V binding and propydium iodide uptake using flow cytometry according to the manufacturer's instructions (Annexin V-Fluorescein Staining Kit; Wako, Osaka, Japan). *In situ* detection of active caspase-3 within virus-infected cells was performed by FITC-DEVD-FMK according to the manufacturer's instructions (GaspGLOW™ Fluorescein Active Caspase-3 Staining Kit; Biovision, Mountain View, CA). Fluorescence for cells was excited with the 488-nm line of an argon laser on an EPICS XL flowcytometer (BECKMAN COULTER Inc., Fullerton, CA). At least 10,000 cells were analyzed for each sample. Apoptosis-positive cells compared with non-infected cells were expressed as a relative percentage of all tested cells. The results were shown as representative data of two repeated experiments.

### Progeny virus production

Cells were infected with IAV at an MOI of 1–2 PFU per cell at 34°C for 1 h. To evaluate progeny virus production, the supernatant at 24 h postinfection was harvested and treated with acetylated trypsin (5 µg/ml) to activate viral infection at 34°C for 1 h. Quantitation of virus titers in supernatants was performed by a plaque assay as described previously [15]. The virus titers (± standard deviation) in the supernatant were expressed as a relative percentage of that in a control medium and were shown as an average of three experiments.

### Virus replication

Cells were infected with IAV at an MOI of 5×10^−3^ PFU per cell at 34°C for 1 h. To evaluate virus multiple replications, the infected cells were maintained in a serum-free medium containing the indicated concentrations of CyA or VAD in the presence of acetylated trypsin (2 µg/ml), which was required for IAV multiple replications. The supernatant at 28 h postinfection was harvested. Quantitation of virus titers in supernatants was performed by a plaque assay as described previously [15]. The virus titers (± standard deviation) in the supernatant were expressed as a relative percentage of that in a control medium and were shown as an average of three experiments.

### Detection of mitochondrial membrane potential

Mitochondrial membrane potential was analyzed by JC-1 (Biovision, Mountain View, CA). Cells were infected with IAV at an MOI of 1–2 PFU per cell at 34°C for 1 h and maintained in a medium containing samples supplemented with 5% FBS at 34°C for 24 h. Cells were harvested by treatment with 0.125% trypsin and incubated with JC-1 (3 µg/ml) for 15 min at room temperature in darkness. As a control, a medium without any samples supplemented with 5% FBS was used. After centrifugation, cells were subjected to quantification by using a Wallac 1420 ARVOsx multi-label counter (PerkinElmer, Waltham, MA, USA). Excitation/emission filter sets of 550 nm/590 nm (red), corresponding to the J-aggregate form of JC-1, and 485 nm/535 nm (green), corresponding to the monomeric form of JC-1, were used. The JC-1 ratio of each cell was calculated as red value per green subtracted by respective fluorescent values of no cells and was shown as a relative percentage of non-infected cells. The results were shown as an average of three experiments.

### Confocal microscopy

Cells were infected at an MOI of 5–10 PFU per cell at 37°C for 30 min. Cells were maintained in a medium containing samples supplemented with 2% FBS at 37°C for 7 h. Then the infected cells were fixed and permeabilized with cold methanol for 30 sec and incubated with rabbit anti-AIF polyclonal antibody (Biovision, Mountain View, CA) and mouse anti-IAV NP monoclonal antibody (4E6). The nucleus in cells was stained with 4′, 6-diamidino-2-phenylindole dihydrochloride (DAPI) (DOJINDO LABORATORIES, Kumamoto, Japan). Fluorescence for samples was observed with an LSM 510 confocal microscope (Carl Zeiss Inc., Thornwood, N.Y.) at a magnification of 400.

### Small interfering RNA of AIF

All siRNAs were synthesized by a Silencer™ siRNA Construction Kit (Ambion, Austin, TX) according to the manufacturer's instructions. SulCOS1 cells at 30% confluency were transfected with 10 nM effective siRNA specific for human AIF (AIF1; 5′-AACTTGTTCCAGCGATGGCAT-3′), ineffective siRNA specific for mouse AIF (AIF2; 5′-AAATGCAGAACTCCAAGCACG-3′) [16], [17], and control siRNA specific for green fluorescence protein (GFP1; 5′-ACTGGAGTTGTCCCAATTCT-3′) using TransIT-TKO (Panvera, Madison, WI) at 37°C for 48 h. Effect of RNA interference was confirmed by immunoblotting with rabbit anti-AIF polyclonal antibody and mouse anti-glyceraldehyde dehydrogenase (GAPDH) monoclonal antibody (CHEMICON International Inc., Temecula, CA).

## Results

**Figure 1:**
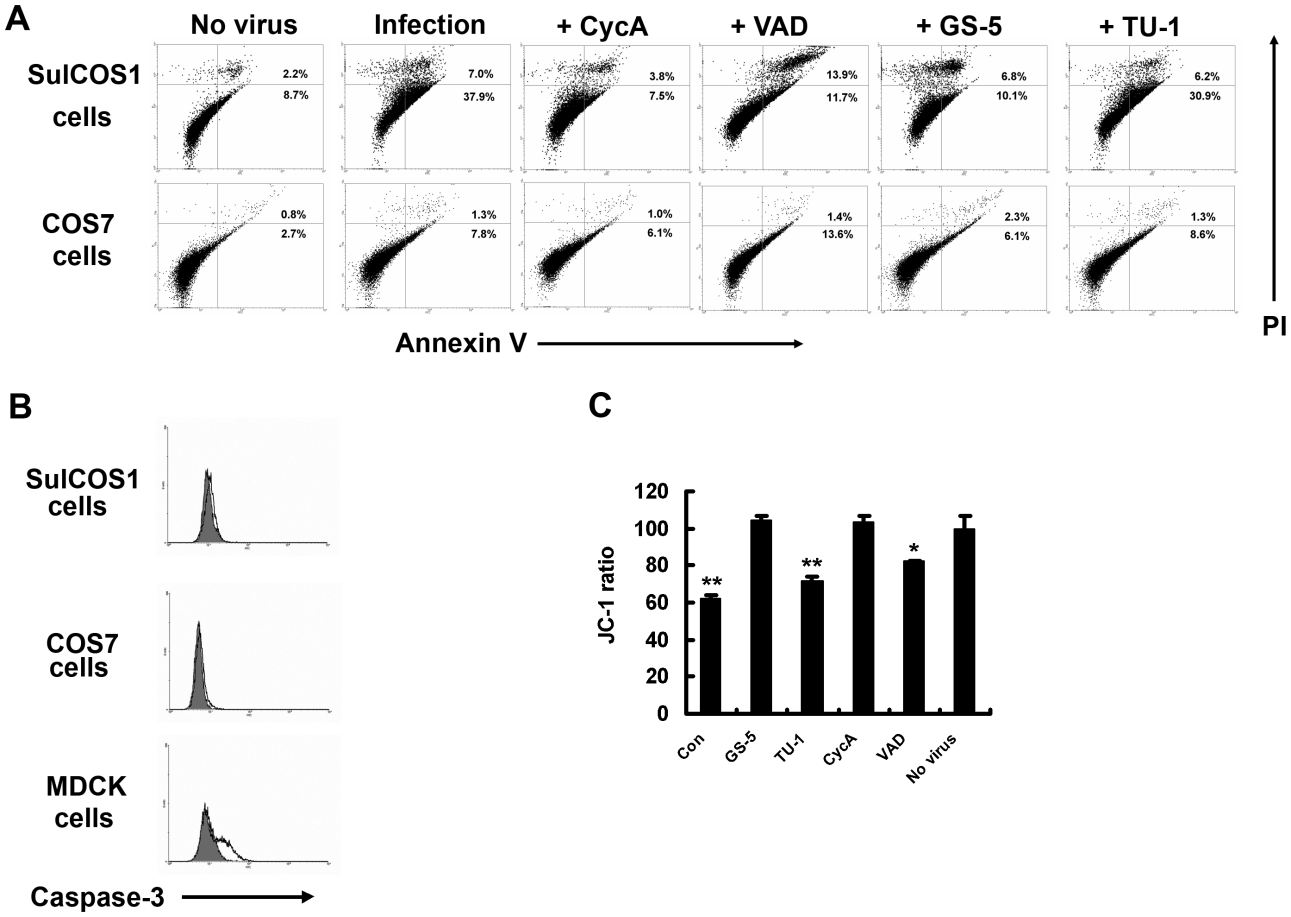
Inhibitory effects of CycA and GS-5 treatment on virus-induced apoptosis. Virus-infected SulCOS1 cells were incubated at 34°C for 24 h. A, Inhibitory effects of CycA and GS-5 on virus-induced apoptosis at 24 h postinfection. CyA and VAD were used at 20 µM and 50 µM, respectively. B, Detection of active caspase-3 in virus-infected cells. MDCK cells were used as a positive control of caspase-3 activation. Gray-painted histograms and lined histograms were results of non-infected cells and infected cells, respectively. The results shown in (A) and (B) are representative data of two repeated experiments. C, Inhibitory effects of CycA and GS-5 on mitochondrial membrane potential loss in virus-infected cells. The JC-1 ratio of each cell was calculated as red value per green subtracted by respective fluorescent values of no cells and is shown as a relative percentage of non-infected cells. The results (± standard deviation) shown are average values of three experiments. Student's t-test was used for statistical analysis compared to no virus in (C). *. *p*<0.05; **, *p*<0.01.

We checked caspase-3 activity, a main pathway of IAV-induced apoptosis, in infected cells. Simultaneously, annexin V staining was used as another indicator of virus-induced apoptosis. Annexin V detects translocation of phosphatidylserine to the outer leaflet of the plasma membrane by apoptosis. Sulfatide-deficient COS7 cells showed no virus-induced apoptosis, whereas sulfatide-enriched SulCOS1 cells became dramatically susceptible to apoptosis (Figure 1A). However, it is surprising that SulCOS1 cells had no active caspase-3, regardless of apoptosis induction (Figure 1B). Caspase-8 and -9 activities have often been used as major indicators of IAV-induced apoptosis [18]–[20]. We also measured caspase-8 and -9 activities of IAV-infected cells. SulCOS1 cells had activations of both caspases, but COS7 cells did not. For MDCK cells, caspase-9 showed a higher level of activation than that of caspase-8, the opposite results to those for SulCOS1 cells (Figure S1). It is thought that sulfatide expression contributes to activations of caspase-8 and -9. Since caspase-9 activity is enhanced via mitochondria by a part of the apoptosis-inducing signals such as activation of caspase-8, a high level of activation of caspase-8 may be correlated with caspase-9 activation. Cyclosporin A (CycA), an inhibitor of the mitochondrial permeability transition pore [21], inhibited apoptosis in SulCOS1 cells (Figure 1A). Conversely, Z-VAD-FMK (VAD), a pan-caspase inhibitor [22], did not inhibit apoptosis more efficiently than CycA (Figure 1A). These results suggest that IAV-induced apoptosis in SulCOS1 cells is derived from activation of a caspase-3-independent pathway associated with mitochondria and sulfatide expression. To determine whether virus-induced apoptosis in SulCOS1 cells is mediated by mitochondria, we analyzed the mitochondrial membrane potential using JC-1 [21], a mitochondrial-specific lipophilic cationic fluorescence dye. Virus infection reduced mitochondrial membrane potential in SulCOS1 cells (Figure 1C). CycA stabilized the potential in SulCOS1 cells, but VAD did not (Figure 1C). These different effects of CycA and VAD indicate that SulCOS1 cells underwent caspase-3-independent apoptosis through mitochondria. Our previous study indicated that a mouse anti-sulfatide monoclonal antibody (GS-5) inhibited IAV replication in SulCOS1 cells [10]. GS-5 suppressed the decline of potential and induction of apoptosis in SulCOS1 cells, but a mouse anti-Gb_3_Cer monoclonal antibody (TU-1) did not (Figure 1A).

**Figure 2:**
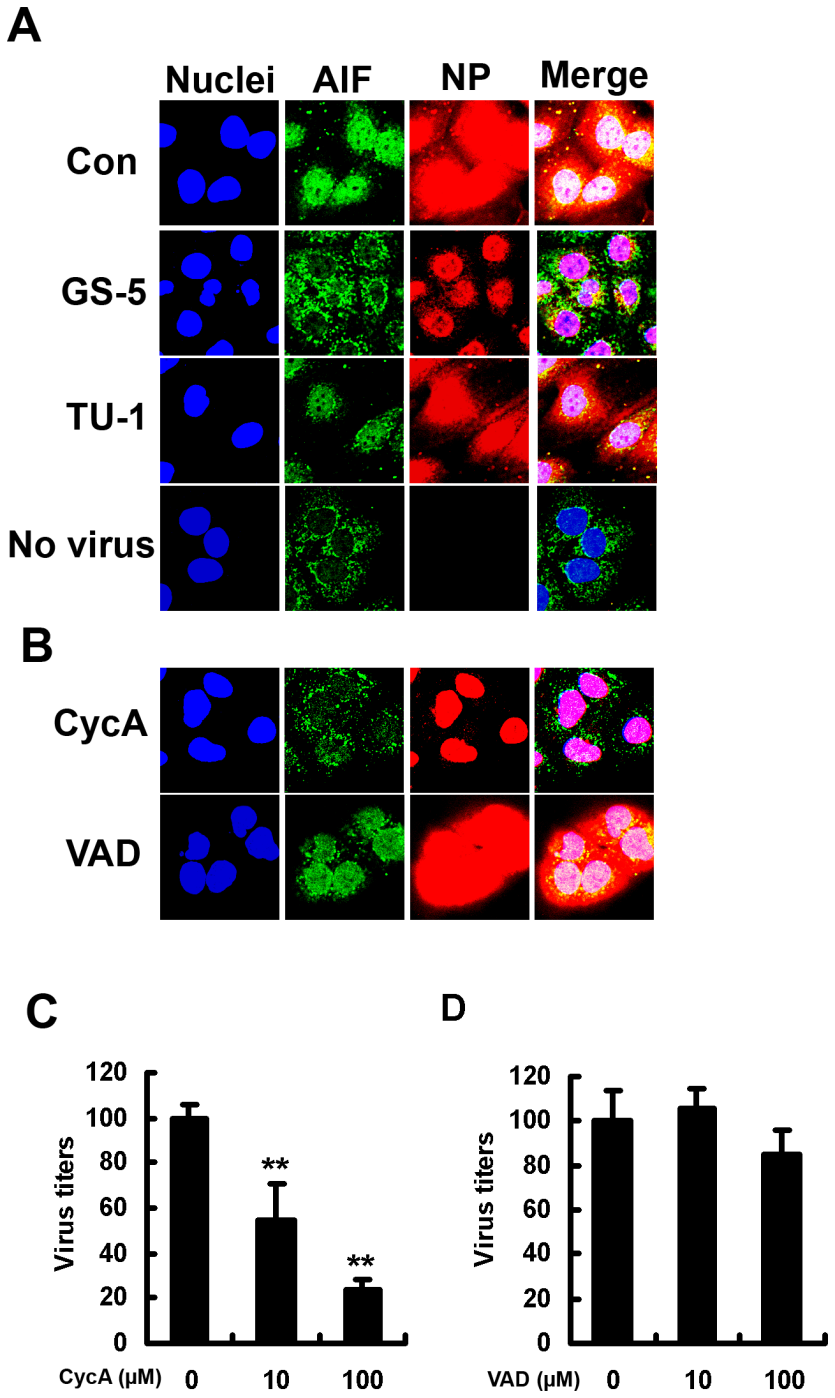
Effects of GS-5 and CyA on AIF and viral NP localization and on virus replication. A–B, Effects of GS-5 and CycA on nuclear translocation of AIF and nuclear export of viral NP in IAV-infected cells. Virus-infected SulCOS1 cells were maintained in a medium without (Con) or with GS-5, TU-1, CycA (50 µM), or VAD (100 µM) at 37°C for 7 h. After fixation of cells, AIF and viral NP were immunostained in green and red, respectively. Nuclei were stained in blue. C and D, Inhibitory effects of CycA treatment (C) and VAD treatment (D) on virus replication of SulCOS1 cells at 28 h postinfection. The virus titers (± standard deviation) in supernatants are expressed as a relative percentage of those with no CycA or VAD treatment and are average values of three experiments. Student's t-test was used for statistical analysis compared to no CycA or VAD treatment in (C) and (D). **, *p*<0.01.

Translocation of AIF from mitochondria to the nucleus is known as an indicator of caspase-3-independent apoptosis through mitochondria [13]. Moreover, apoptosis helps progeny virus formation and replication of IAV through increased viral NP export from the nucleus to the cytosol [6], [10]. Translocation of the newly synthesized viral NP is required for infectious progeny virus formation. We therefore observed translocation of AIF and viral NP export under a confocal microscope. Translocation of AIF to the nucleus caused by virus infection was clearly observed in SulCOS1 cells. This is evidence of caspase-3-independent apoptosis in IAV-infected SulCOS1 cells. We previously showed that suppression of apoptosis by GS-5 treatment resulted in a delay of viral NP export from the nucleus. Treatment of SulCOS1 cells with GS-5 significantly suppressed both translocation of AIF to the nucleus and NP export to the cytosol, whereas treatment of SulCOS1 cells with TU-1 did not suppress either of these (Figure 2A). Treatment of SulCOS1 cells with CycA inhibited NP export to the cycosol, whereas treatment of the cells with VAD did not (Figure 2B). Treatment of SulCOS1 cells with CycA resulted in much greater suppression of progeny virus production and replication (Figure 2C) than did VAD treatment (Figure 2D). Ineffectiveness of VAD treatment further supports activation of a caspase-3-independent apoptosis pathway in IAV-infected SulCOS1 cells. Taken together, the results indicate that sulfatide-mediated caspase-3-independent apoptosis in IAV-infected SulCOS1 cells plays an important role in efficient viral NP export from the nucleus to the cytosol. Enhancement of viral NP export leads to increased virus replication.

**Figure 3:**
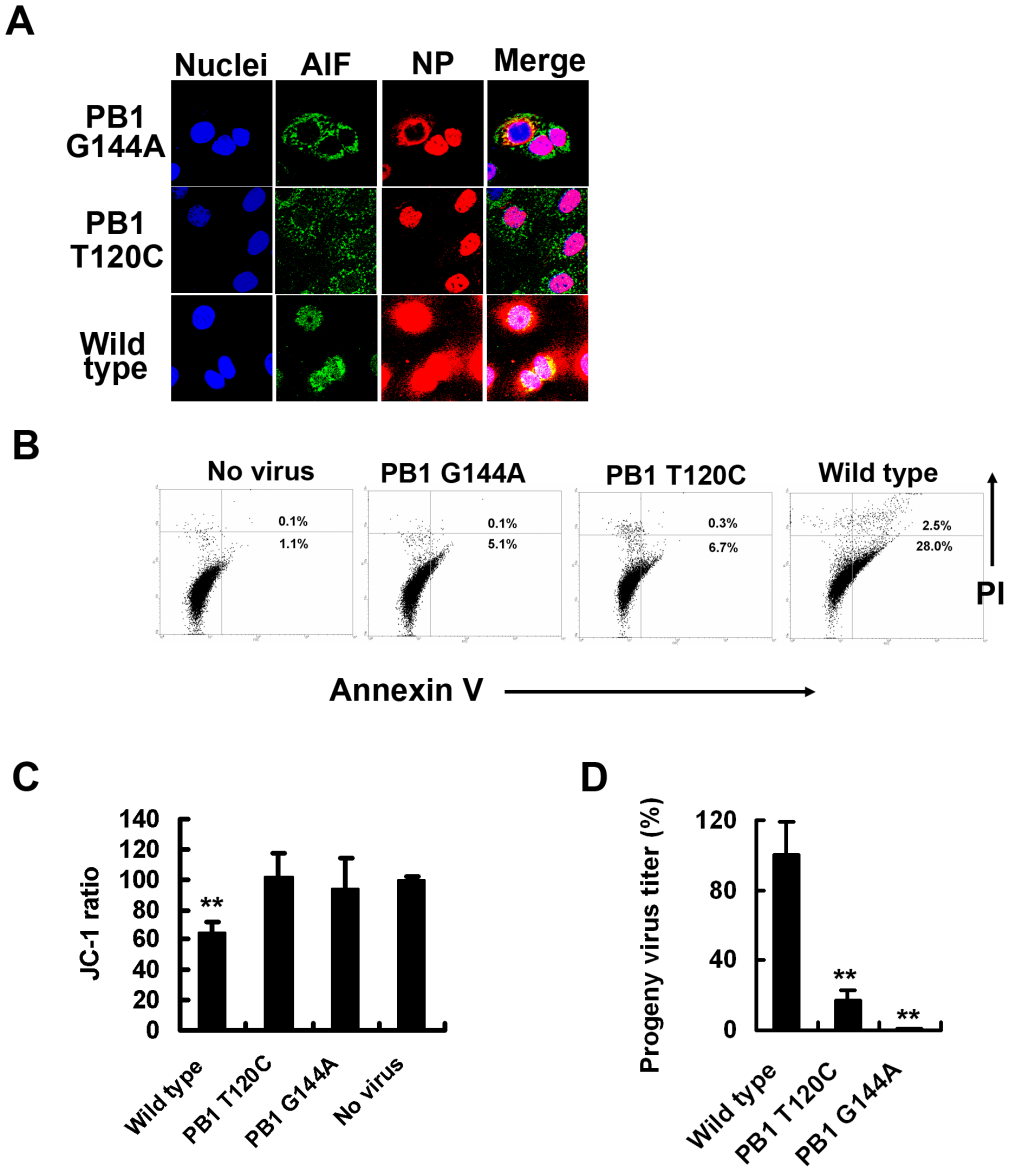
PB1-F2 mediates induction of caspase-3-independent apoptosis in SulCOS1 cells. SulCOS1 cells were infected with two recombinant PB1-F2-deficient mutant viruses (PB1 G144A and PB1 T120C) and wild-type virus. A, Fluorescent staining of AIF, NP, and nuclei at 7 h postinfection as described in the legend of Figure 2. B–D, SulCOS1 cells were infected with the respective virus as described in the Materials and Methods section. Inhibition of virus-induced apoptosis (B), mitochondrial membrane potential loss (C), and progeny virus production (D) in cells infected with PB1-F2-deficient viruses at 24 h postinfection. The progeny virus titers (± standard deviation) in supernatants are expressed as a relative percentage of those of wild-type virus and are average values of three experiments. Student's t-test was used for statistical analysis compared to no virus in (C) and compared to wild-type virus in (D). **, *p*<0.01.

**Figure 4:**
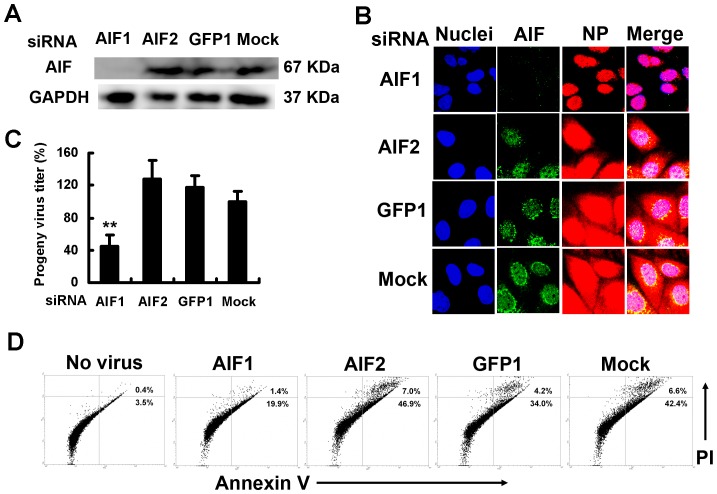
Inhibition of virus-induced apoptosis and progeny virus production in SulCOS1 cells by siRNA against AIF. SulCOS1 cells were transfected with efficient small interfering RNA (siRNA) against AIF (AIF1) or inefficient siRNA (AIF2 and GFP1) as a control. Mock means transfection without siRNA into infected cells. A, AIF and GAPDH were immunoblotted at 48 h posttransfection. B–D, After transfection with siRNA for 48 h, the cells were infected with the virus as described in the Material and Methods section. B, Fluorescent staining of AIF, NP, and nuclei at 7 h postinfection as described in the legend of Figure 2. C, Inhibitory effect of AIF1 on progeny virus production at 24 h postinfection. The progeny virus titers (± standard deviation) in supernatants are expressed as a relative percentage of those of mock treatment and are average values of three experiments. Student's t-test was used for statistical analysis compared to mock treatment in (C). **, *p*<0.01. D, Inhibitory effect of AIF1 on virus-induced apoptosis at 24 h postinfection. The results shown in (D) are representative data of two repeated experiments.

PB1-F2, a frame-shift protein from the viral RNA polymerase subunit PB1 gene of IAV, mediates virus-induced apoptosis through mitochondrial membrane potential loss [23]. To determine whether PB1-F2 contributed to caspase-3-independent apoptosis in IAV-infected SulCOS1 cells, we generated two PB1-F2-deficient IAVs using a reverse genetics method. One virus (PB1 T120C) expressed the PB1 gene with a T-to-C substitution at nucleotide 120, introducing an alteration in the proposed Met start codon to Thr without affecting the standard reading frame. The other (PB1 G144A) expressed the PB1 gene with a G-to-A substitution at nucleotide 144, introducing a stop codon after translation of only eight residues of PB1-F2, and a Met-to-Ile substitution in PB1 at position 40. Infection of SulCOS1 cells with PB1-F2-deficient virus significantly delayed viral NP export to the cytosol (Figure 3A) and diminished apoptosis concomitant with no translocation of AIF to the nucleus, compared to recombinant wild-type virus (Figure 3B). The results indicate that PB1-F2 can function as an inducer of caspase-3-independent apoptosis and therefore invokes efficient NP export in SulCOS1 cells. PB1-F2-deficient virus did not reduce the mitochondrial potential in SulCOS1 cells (Figure 3C). Besides, when SulCOS1 cells were infected with the two PB1-F2-deficient viruses, each progeny virus titer in the supernatant at 24 h postinfection was decreased to less than 17% of that in the case of wild-type virus infection (Figure 3D). These results indicate that PB1-F2 can induce sulfatide-associated caspase-3-independent apoptosis through AIF translocation concomitant with destruction of mitochondria membrane potential and enhance the progeny virus production and replication in virus-infected SulCOS1 cells. To confirm the contribution of AIF to efficient NP export in SulCOS1 cells, we transfected small interfering RNA (siRNA) that targeted AIF mRNA [16], [17] and evaluated the degree of NP export and progeny virus production. Effective siRNA AIF1 (Figure 4A) significantly suppressed NP export (Figure 4B), progeny virus production (Figure 4C), and virus-induced apoptosis (Figure 4D) in comparison with the effects of ineffective siRNA AIF2 and GFP1 (targeting green fluorescent protein). The results suggest contribution of the caspase-3-independent apoptosis process through AIF to efficient virus replication in SulCOS1 cells.

## Discussion

Here, we investigated the function of sulfatide in IAV-induced apoptosis. A recent common concept of IAV-induced apoptosis is both the caspase-dependent pathway through capsase-3 activation and the caspase-3-independent pathway through AIF [24]. HA on the cell surface membrane is thought to induce nuclear export of vRNP through caspase 3-dependent apoptosis beginning with the Raf/MEK/ERK signal [6], [8]. In SulCOS1 cells, IAV can also induce caspase-3-independent apoptosis through AIF. Sulfatide expression is linked to this process through the mitochondrial membrane potential loss caused by viral PB1-F2 protein. Similar to caspase-dependent apoptosis, this process also promotes progeny virus production and replication because of the increased export of newly synthesized vRNP from the nucleus to the cytosol. Z-VAD-FMK had a slight inhibitory effect on the mitochondrial membrane potential loss (Figure 1C). It is thought that this potential loss is partially caused by caspase-8 activation, and this potential loss may be associated with caspase-9 activation. For SulCOS1 cells, a MEK inhibitor, U0126, completely inhibited IAV replication. On the other hand, a protein kinase C activator (a Raf/MEK/REK activator), 12-O-tetradecanoyl phorbol-13-acetate (TPA), increased IAV replication (Figure S2). Raf/MEK/REK signal was linked to IAV replication in SulCOS1 cells. It is thought that sulfatide expression is associated with activation of the Raf/MEK/REK signal in IAV-infected cells.

Caspase-independent apoptosis has been observed in specified cell types, including several human lymphocyte lines, human respiratory cell lines, and COS cell lines, which are parent cell lines of SulCOS1 cells [25], [26]. Destination of the IAV-induced apoptosis pathway is likely to be dependent on the cell type. Caspase-3 activation is dominant in many types of cells. Since many human respiratory cell lines acquire the ability to invoke caspase-independent apoptosis [26], [27], it is of more interest to evaluate actual IAV-induced apoptosis in respiratory cells of clinical individuals or primary respiratory cell lines.

**Figure 5:**
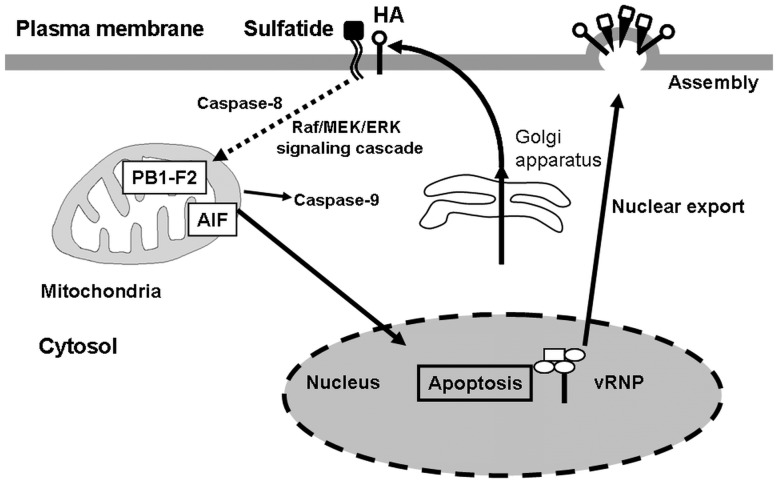
Putative scheme of nuclear export of vRNP invoked by sulfatide-associated apoptosis. Once sulfatide binds with the newly synthesized HA transferred to the cell surface membrane, mitochondrial membrane potential loss is invoked in mitochondria localized with PB1-F2. AIF translocates from mitochondria to the nucleus, followed by induction of apoptosis. Apoptosis enhances nuclear export of vRNP from the nucleus, resulting in promotion of progeny virus production. Since activation of the Raf/MEK/ERK pathway is linked to both surface membrane translocation of the newly synthesized HA and apoptosis induction [8], this pathway is possible between sulfatide binding of HA and mitochondria membrane potential loss. A dotted line is a putative pathway.

Ion selectivity of PB1-F2 as an ion channel shows highly specific susceptibility to cadmium ion [4]. Cadmium also induces caspase-independent apoptosis through mitochondrial membrane potential loss and AIF translocation in normal human lung cells [27]. These reports support the ability of PB1-F2 to mediate caspase-3-independent apoptosis. Treatment of IAV-infected MDCK cells with an anti-sulfatide monoclonal antibody or an anti-HA monoclonal antibody, which blocks the binding of IAV and sulfatide, resulted in significant reduction in IAV replication and accumulation of the viral NP in the nucleus [10]. Taken together, our findings lead to the following three conclusions; i) association of sulfatide with cell surface-delivered HA induces the caspase-3-independent pathway, and sulfatide-associated apoptosis invokes nuclear export of vRNP, ii) IAV infection of sulfatide-enriched cells induces caspase-3-independent apoptosis through AIF, iii) viral PB1-F2 in sulfatide-enriched cells mediates caspase-3-independent apoptosis through AIF (Figure 5). It has been suggested that apoptosis increases the diffusion limit of nuclear pores to allow passive diffusion of larger proteins, directly or indirectly through active caspases [6]. Apoptosis through AIF and PB1-F2 may also promote the nuclear export of vRNP complexes by increase in the diffusion limit of nuclear pores.

IAV causes necrosis in some cell lines such as human epithelial colon carcinoma COCA-2 cells and human bronchial H1299 cells. Necrotic plasma membrane permeabilization enables annexin V to bind to intracellular phosphatidylserine and also enables propidium iodide to stain the intracellular nucleus [28], [29]. In the present study, since apoptosis was mainly measured by annexin V staining and propidium iodide staining, these stainings might mean sulfatide-associated necrosis. However, this apoptosis in SulCOS1 cells was characterized by activation of caspase-8 and -9 (Figure S1), mitochondrial membrane potential loss (Figure 1C and 3C), and nuclear localization of AIF (Figure 2A, 3A, and 4B). Therefore, IAV-induced cell death in SulCOS1 cells is thought to be apoptosis rather than necrosis.

Although AIF expression was effectively knocked down, progeny virus production was still more than 40% compared to mock treatment (Figure 4). Our previous study showed a very low level of virus production from IAV-infected COS7 cells that were ineffective in nuclear export of NP [10]. Nuclear export of NP is not necessarily completely dependent on apoptosis associated with AIF, PB1-F2, and sulfatide. Some of the vRNP complexes in the nucleus may be subjected to passive diffusion to the cytosol or to an unidentified transport mechanism, independently of apoptosis. This leak of vRNP complexes in the nucleus leads to a low level of progeny virus production.

Further investigation of sulfatide-associated caspase-3-independent apoptosis will lead to elucidation of an alternative IAV infection mechanism and to the development of a new practical anti-IAV strategy. Since sulfatide-associated apoptosis remains poorly defined, it may be interesting to pay attention to the fact that sulfatide expression functions as an inducer of caspase-3-independent apoptosis. Sulfatide is a universal widespread glycolipid in many mammalian tissues and cells [30]. Therefore, further investigation of sulfatide-associated apoptosis should also provide new insights into the wide spectrum of anti-virus, anti-bacteria, and anti-tumor strategies based on regulation of apoptosis.

## Supporting Information

Figure S1 **Sulfatide expression enhances caspase-8 and -9 activities in virus-infected cells.** SulCOS1 cells, COS7 cells, and MDCK cells (1.25×10^4^ cells/well) in 96-well culture plates were washed with PBS and infected with A/WSN/33 (H1N1) strain in 100 µl of a serum-free medium [Hybridoma-SFM (SFM), Life Technologies Corp., Carlsbad, CA] at a MOI of 5 pfu/cell for 1 h at 34°C. After washing the cells with PBS, the cells were cultured with 100 µl/well of SFM containing 10% FBS for 24 h at 34°C. One hundred microliters per well of a caspase-8 and -9 assay kit (Promega Corp., Madison, WI) was added to the cell culture plate. After incubation for 30 min at room temperature, luminescent intensities showing caspase activities were measured by using a GloMax™ 96 Microplate Luminometer (Promega Corp., Madison, WI). Caspase activities (%) were expressed as a relative percentage of each caspase activity in non-infected cells. Standard deviations were calculated by three independent experiments. Student's *t*-test was used for statistical analysis compared to non-infected cells. **, *p*<0.01. Empty column, non-infected cells; Closed column, infected cells.
(TIF)

Figure S2 **Effect of U0126 on IAV replication in SulCOS1 cells.** SulCOS1 cells in a 24-well tissue culture plate (0.5×10^5^ cells/well) were infected with a MOI of 0.01 of A/WSN/33 (H1N1) strain in 250 µl/well of SFM at 37°C for 30 min. After washing the cells with PBS, the cells were cultured at 34°C in 500 µl/well of SFM containing 2 µg/ml acetylated trypsin in the presence of DMSO (0.5%), MEK inhibitor U0126 (25 µM), or protein kinase C activatior 12-O-tetradecanoyl phorbol-13-acetate (TPA, 100 ng/ml). At 24 h postinfection, virus titers in the supernatant were measured by a focus assay using low-viscosity (Avicel) overlay medium [31]. Virus titers (standard error bars) are expressed as a relative percentage of those with DMSO and are average values of three experiments. Results of U0126 were undetectable levels, less than 10 focus forming units. Virus titers (%) in supernatants are expressed as a relative percentage of DMSO treatment. Standard deviations were calculated by three independent experiments. Student's *t*-test was used for statistical analysis compared to non-infected cells. *, *p*<0.05; **, *p*<0.01.
(TIF)
