# Supplementary figures and images for: Sulfatide Regulates Caspase-3-Independent Apoptosis of Influenza A Virus through Viral PB1-F2 Protein
Source: PLoS One. 2013 Apr 4;8(4):e61092. doi: 10.1371/journal.pone.0061092 (PMC3617187; doi:10.1371/journal.pone.0061092)

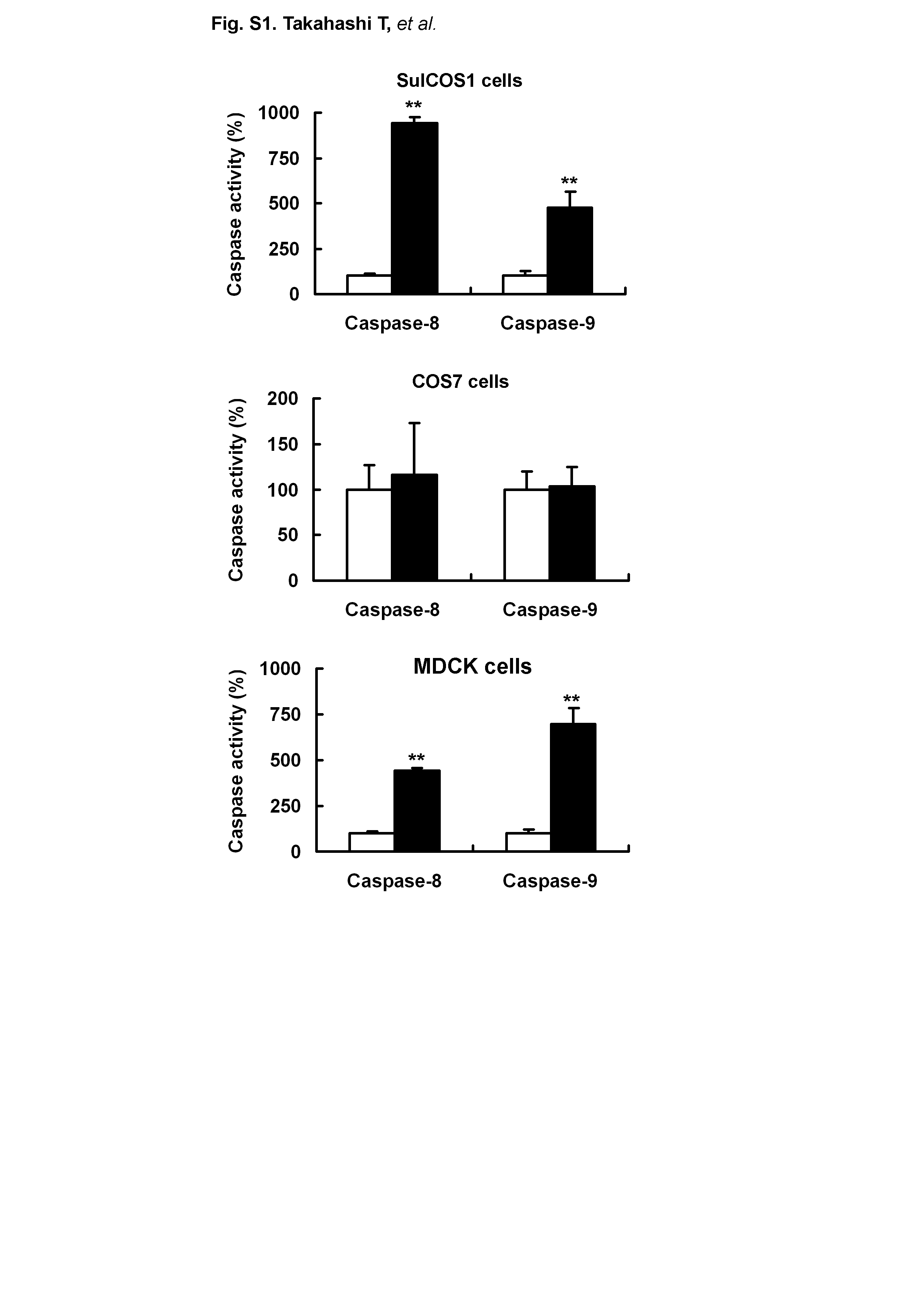

Supplement: Figure S1 — Sulfatide expression enhances caspase-8 and -9 activities in virus-infected cells. SulCOS1 cells, COS7 cells, and MDCK cells (1.25×10^4^ cells/well) in 96-well culture plates were washed with PBS and infected with A/WSN/33 (H1N1) strain in 100 µl of a serum-free medium [Hybridoma-SFM (SFM), Life Technologies Corp., Carlsbad, CA] at a MOI of 5 pfu/cell for 1 h at 34°C. After washing the cells with PBS, the cells were cultured with 100 µl/well of SFM containing 10% FBS for 24 h at 34°C. One hundred microliters per well of a caspase-8 and -9 assay kit (Promega Corp., Madison, WI) was added to the cell culture plate. After incubation for 30 min at room temperature, luminescent intensities showing caspase activities were measured by using a GloMax™ 96 Microplate Luminometer (Promega Corp., Madison, WI). Caspase activities (%) were expressed as a relative percentage of each caspase activity in non-infected cells. Standard deviations were calculated by three independent experiments. Student's t-test was used for statistical analysis compared to non-infected cells. **, p<0.01. Empty column, non-infected cells; Closed column, infected cells. (TIF) [file pone.0061092.s001.tif]

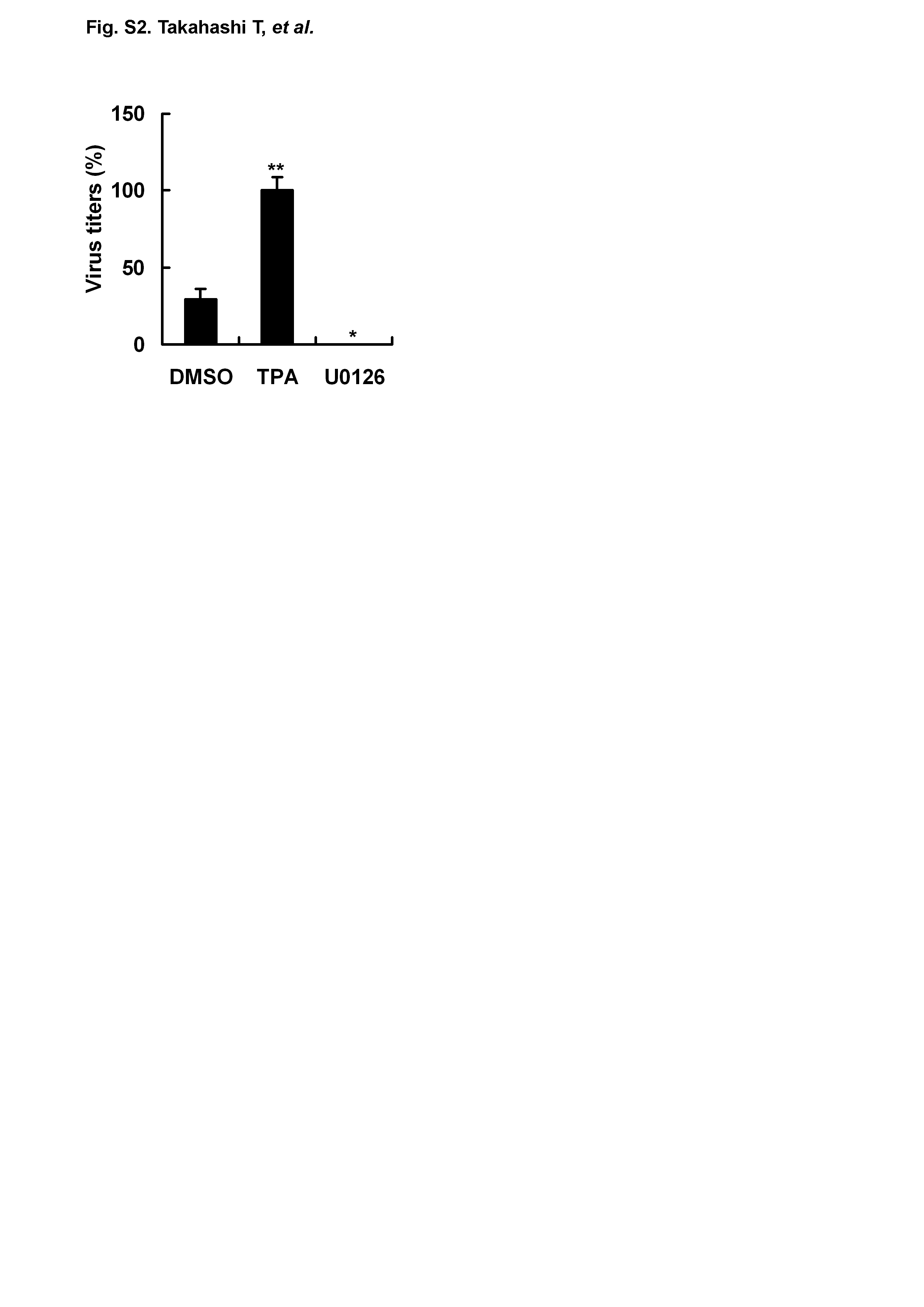

Supplement: Figure S2 — Effect of U0126 on IAV replication in SulCOS1 cells. SulCOS1 cells in a 24-well tissue culture plate (0.5×10^5^ cells/well) were infected with a MOI of 0.01 of A/WSN/33 (H1N1) strain in 250 µl/well of SFM at 37°C for 30 min. After washing the cells with PBS, the cells were cultured at 34°C in 500 µl/well of SFM containing 2 µg/ml acetylated trypsin in the presence of DMSO (0.5%), MEK inhibitor U0126 (25 µM), or protein kinase C activatior 12-O-tetradecanoyl phorbol-13-acetate (TPA, 100 ng/ml). At 24 h postinfection, virus titers in the supernatant were measured by a focus assay using low-viscosity (Avicel) overlay medium [31]. Virus titers (standard error bars) are expressed as a relative percentage of those with DMSO and are average values of three experiments. Results of U0126 were undetectable levels, less than 10 focus forming units. Virus titers (%) in supernatants are expressed as a relative percentage of DMSO treatment. Standard deviations were calculated by three independent experiments. Student's t-test was used for statistical analysis compared to non-infected cells. *, p<0.05; **, p<0.01. (TIF) [file pone.0061092.s002.tif]
